# SimPep and OP-AND: A deep learning framework and curated database for predicting osteogenic peptides

**DOI:** 10.1371/journal.pcbi.1013422

**Published:** 2025-08-29

**Authors:** Maryam Ghobakhloo, Zahra Ghorbanali, Fatemeh Zare-Mirakabad, Roya Abbaszadeh, Mohammad Taheri-Ledari, Bahman Zeynali

**Affiliations:** 1 Department of Cell and Developmental Biology, School of Biological Sciences, College of Science, University of Tehran, Tehran, Iran; 2 Computational Biology Research Center (CBRC), Department of Mathematics and Computer Science, Amirkabir University of Technology, Tehran, Iran; 3 Department of Biology, Philipps-University Marburg, Marburg, Germany; 4 Department of Bioinformatics, Institute of Biochemistry and Biophysics (IBB), University of Tehran, Tehran, Iran; 5 Department of Cell and Developmental Biology, School of Biological Sciences, College of Science, University of Tehran, Tehran, Iran; KU: The University of Kansas, UNITED STATES OF AMERICA

## Abstract

Bone health is a growing concern in aging populations, and bioactive peptides in dairy products offer a promising approach to preventing bone-related diseases. However, the lack of a public database for osteogenic peptides (OPs) has limited the computational detection efforts. In this work, we introduce OP-AND, a curated public database of osteogenic peptides. We also propose a novel hypothesis that peptides derived from proteins involved in osteoclast formation may serve as non-osteogenic. Considering the limited availability of OP data, we present SimPep, a deep learning framework that achieves 86.87% accuracy and 76.88% area under receiver-operating characteristic curve score using five-fold cross-validation. SimPep’s performance is further evaluated on external datasets, and a pipeline is introduced to select potential OPs for experimental studies. The camel milk alpha s1-casein peptide ‘MKLLILTCLVAVALARPKYPLRYPEVF’ is highlighted as a top candidate for future exploration. The OP-AND database is available in https://github.com/CBRC-lab/SimPep_and_OP-AND.

## Author summary

Certain small protein fragments, called peptides, found in dairy products have shown potential to support bone growth and prevent diseases such as osteoporosis. However, researchers currently lack a dedicated and organized database to study these bone-strengthening peptides computationally. In this work, we introduce OP-AND, the first publicly available database focused on peptides with bone-forming potential. To facilitate peptide discovery, we also develop a deep learning model named SimPep, designed to predict whether a given peptide exhibits osteogenic properties. To our knowledge, this represents the first comprehensive effort in this area and lays the foundation for future research in computational osteogenic peptide discovery. Our model demonstrates strong performance across various experiments and helps identify promising candidates, such as a peptide derived from camel milk, for further laboratory testing. To support the broader scientific community, we make both the OP-AND database and the SimPep model publicly accessible on GitHub. Additionally, we provide a companion tool, SimPep-App, which enables osteogenicity analysis of peptides.

## 1. Introduction

Bone tissue is a multifaceted and vital human body component. Beyond providing structural support, bones act as the calcium repositories, regulate mineral homeostasis, and play a crucial role in hematopoiesis. These essential functions, highlighting the importance of addressing skeletal disorders [1,2], including osteoporosis. Osteoporosis is considered as a public health concern in the rapidly aging population, which can be contributed to an increase in bone fractures and associated mortality. Current osteoporosis treatments primarily focus on reducing bone resorption but are insufficient in restoring lost bone structure [3].

The use of anabolic medications and nutritional supplements combination is a promising strategy in addressing this condition, alongside advancements in bone tissue engineering [4]. The efficacy of tissue engineering relies on integrating bioactive molecules necessary for promoting optimal osteogenic differentiation. In this context, proteins and peptides derived from dietary resources, particularly milk, demonstrate a wide range of biological activities [5]. Among these, the capacity for tissue protection, stimulation of osteoblast differentiation and proliferation stand out, offering considerable potential in the realm of bone tissue regeneration [6].

Research in bone tissue engineering and the use of bioactive peptides (BAPs) has expanded significantly in recent years [7–9]. Numerous peptides have demonstrated bone health-promoting effects, generating considerable interest in their potential for developing new therapies to treat bone-related disorders such as osteoporosis, bone fractures, and other bone diseases [9,10]. BAPs present a more cost-effective and safer alternative to traditional protein-based therapies. They also exhibit fewer side effects and lower toxicity in the human body, making them a promising candidate for future therapeutic interventions in bone health [11].

Currently, researchers have created several comprehensive databases of BAPs, encompassing antimicrobial peptides [12,13], anti-cancer peptides [14], peptide/HLA complexes [15] and more. These databases have been instrumental in designing sophisticated algorithms that enable the rapid and precise prediction of BAPs [15–17]. However, despite experimental reports mentioning osteogenic peptides (OPs) in various studies [18,19], to the best of our knowledge, no dedicated database for OPs has been established.

In this regard, an in vitro study [20] was conducted to investigate the osteogenic potential of peptides derived from camel milk. The study demonstrated that casein hydrolysates smaller than 10 kDa could stimulate osteogenic activity and promote osteogenesis in mesenchymal stem cells [20]. Casein was enzymatically hydrolyzed using the chymotrypsin, and ultrafiltration membranes were employed to remove non-hydrolyzed proteins and large peptides. The osteo-inductive capacity of the resulting peptide fraction was evaluated using alizarin red staining, which revealed significantly enhanced mineralization in fraction lacking large peptides (p < 0.05). These findings were further supported by real-time PCR analysis of osteogenic markers such as RUNX-2 and alkaline phosphatase. However, due to the high cost and time-intensive nature of mass spectrometry, the study did not identify the specific peptide sequences responsible for the observed osteogenic effects [21].

Although computational approaches offer a promising and cost-effective solution for osteogenic peptide detection (OPD), their effectiveness heavily depends on the availability of high-quality, curated data. Unfortunately, the absence of a dedicated OP database has significantly limited progress in this field.

To address the lack of curated data, a previous study [22] manually curated a set of 82 OPs that had been experimentally validated in the literature [22]. In parallel, a computational model was defined to predict OPs, employing a profile hidden Markov model (pHMM). This approach was chosen due to the limited size of available OPs samples [23]. For model training, a set of non-osteogenic peptides (non-OPs) was compiled from random proteins with no known association with osteogenic processes, including ELH1_APLCA, BAG6_HUMAN, and CCL11_HUMAN, retrieved from UniProt [24]. Enzymatic cleavage sites within these proteins were predicted using PeptideCutter [25] with chymotrypsin and trypsin, as described in [20].

The pHMM model achieved a sensitivity of 62%, precision of 52%, and an F1-score of 56% in identifying OPs [22]. In the next step, the model was specifically applied to peptides derived from camel milk casein proteins to explore their potential osteogenic activity. Peptides were generated using PeptideCutter and subsequently analyzed by the pHMM model to identify potential OPs. For experimental validation, two of the highest-scoring peptides were selected as positive controls, and one of the lowest-scoring peptides was chosen as a negative control. While the negative control was correctly identified as non-OP, the positive control peptides unexpectedly failed to exhibit osteogenic properties. This outcome highlights the limitations of both the model and the dataset used for training. In particular, the random selection of non-OPs from unrelated proteins may not provide a biologically meaningful negative class, potentially affecting model performance.

Two major challenges in advancing computational solutions for OPD are the limited availability of experimentally validated OPs and the absence of reliable information on non-OPs. These constraints significantly hinder the effective application of advanced techniques, such as deep learning (DL), in improving OPD prediction accuracy. This paper aims to tackle these challenges as follows:

- Publish a publicly available, comprehensive database of OPs, named OP-AND (Osteogenic Peptide – Abbas Nowzari Dalini) in honor of our late co-author, Abbas Nowzari Dalini, who sadly passed away during this study due to cancer. This database compiles experimentally validated OPs from the literature, including the 82 peptides identified in our previous study [22] based on published evidence prior to 2022, along with 26 newly discovered OPs collected between 2022 and 2024, making it a more robust and up-to-date resource.
- Propose the hypothesis that peptides derived from proteins involved in osteoclast differentiation may serve as non-OPs. Since these proteins primarily contribute to bone resorption without directly influencing osteogenic or bone morphogenetic properties, peptides derived from them can be classified as non-OPs.
- Develop a framework called SimPep to define a DL approach for addressing the OPD problem using OPs extracted from the OP-AND database and non-OPs constructed based on our hypothesis.

We introduce SimPep, a seven-step framework designed to enable OPD prediction using a DL-based approach. In step 1, known OP and non-OP peptides are divided into training and test sets. Step 2 involves a comparative analysis of diverse peptide representations, including biologically motivated sequence descriptors and contextual embeddings generated by pre-trained masked language models (MLMs), to identify the most informative encoding strategy for OPD prediction. Due to the limited number of available OPs and non-OPs, training a traditional DL model directly for OPD prediction is not feasible. To address this challenge, SimPep reformulates OPD as an osteogenic peptide similarity (OPS) classification problem. In this problem, the main goal is to distinguish between peptide pairs based on their similarity in osteogenic potential.

In Step 3, a balanced training dataset for OPS classification problem is constructed from the training set in the first step defined for OPD prediction by creating positive and negative pair peptides. Positive pairs consist of peptides with similar osteogenic properties, while negative pairs contain peptides with distinct properties. This pairing addresses the limited data in the OP and non-OP sets by creating more training samples through the generation of the both intra- and inter-set pairs. This approach effectively increases the dataset size for training a DL model to predict OPS classification.

In Step 4, we introduce SimPep-Net, a deep learning architecture based on a siamese neural network (SNN), specifically designed to capture the similarity patterns between peptide pairs in the context of osteogenesis. In Step 5, an iterative training strategy is employed to enhance the generalization capability of SimPep-Net. In Step 6, the model is evaluated using peptide pairs from the test set to assess its ability to identify osteogenic similarity. Finally, in Step 7, each peptide in the test pool is paired with peptides from the training set. The model predicts similarity scores for each pair, and a similarity-aggregated scoring function is applied to assign a probability score to each test peptide, classifying it as either an OP or a non-OP.

The SimPep framework’s effectiveness is evaluated through five-fold cross-validation for OPS and OPD prediction tasks. The framework is assessed with a dataset containing OPs from the OP-AND database and non-OPs from osteoclast genesis proteins, comparing results with a randomly selected non-OP set, according to the previous study [22]. In addition, the framework is trained on older OP set (pre-2022) and tested on newer ones (2022–2024), alongside experimental non-OPs from previous research [22]. A case study on casein types is conducted to identify potential OPs, and a prediction pipeline is proposed to support these findings. Specifically, camel milk alpha s1-casein is analyzed using SimPep, which identifies candidate OPs for further experimental validation.

## 2. Material and methods

In this section, we define the OPD problem and explain the process of extracting and collecting OPs to build the OP-AND database. We also propose the hypothesis that peptides derived from proteins involved in osteoclast differentiation, which are presumed to lack osteogenic properties, can be classified as non-OPs. Furthermore, we introduce a framework, called SimPep, designed to effectively address the OPD problem using a DL model.

### 2.1. Osteogenic peptides detection problem

Each peptide sequence of length n is defined as follows:


$$P=p_{1}\ldots p_{n},\;|P|=n,$$

where $p_{i}$ (for i=1,…,n) represents one of the 20 different amino acids. The OPD problem is defined as follows:

- Input: P as a peptide sequence.
- Output: $\begin{array}{c} \left\{ \;\;\;\begin{array}{cc} 1, & \text{if }\;P\;\text{peptide}\;\text{has}\;\text{ostagenic}\;\text{properties,}\\ 0, & \;\;\;\;\;\;\;\;\;\;\;\;\;\;\;\;\;\;\;\;\;\;\;\;\;\;\;\;\;\;\;\;\;\;\;\;\;\;\;\;\;\;\;\;\;\;\;\;\;\;\;\;\;\;\;\;\;\;\;\text{othetwise}. \end{array}\right. \end{array}$

In this study, the OPD problem is reformulated to OPS classification problem. Then a DL model is built to solve the OPS classification problem. Finally, the trained DL model is used to solve OPD problem. In the following, the OPS classification problem is defined:

- Input: $<P,P^{\prime }>$ as a pair of peptide sequences.
- Output: $\left\{ \begin{array}{c} 1,\;amp;\;\;\;\;\;\;\;\;\;\;\;<P\text{,}{\text{P}}^{\prime }>\in \;\{<\text{OP,OP}>\text{,}<\text{non}-\text{OP,non}-\text{OP}>\}\text{,}\\ 0,\;amp;\;\;\;\;\;\;\;<P\text{,}{\text{P}}^{\prime }>\in \;\{<\text{non}-\text{OP,}\text{ OP}>\text{,}<\text{OP, }\text{non}-\text{OP}>\}.\;\; \end{array}\right.$

The main goal in OPS classification problem is to distinguish between similar and dissimilar peptide pairs based on their osteogenic properties.

### 2.2. Dataset collection

To prepare a comprehensive database of known OPs, called OP-AND (available in https://github.com/CBRC-lab/SimPep_and_OP-AND), the reputable publications such as Nature [26], PubMed [27] and ScienceDirect [28] are searched using the following keywords: **‘**osteogenic peptides’, ‘bioactive peptides’, ‘osteogenesis’, ‘bone differentiation’, ‘bone metabolism’, ‘bone health’, ‘bone regeneration’ and ‘bone healing’ similar to our previous research [22]. This process results in the manual collection of 108 OPs. Among them, 82 OPs were identified from literature published prior to 2022, based on previous study [22], while the remaining 26 OPs are collected from publications between 2022 and 2024. Therefore, the set of OPs is denoted as $𝕆=\{P_{1}^{𝕆},\ldots P_{108}^{𝕆}\}$.

To generate the set of non-OPs, two alternative approaches are explored:

- First, following a previous study [22], we select non-OPs from random proteins [22] that have no known involvement in osteogenesis. This results in a set of 300 peptides, denoted as ${\mathbb{N}}^{\text{r}}=\{P_{\text{1}}^{{\mathbb{N}}^{\text{r}}}\text{,}\ldots P_{\text{300}}^{{\mathbb{N}}^{\text{r}}}\}$.
- Second, based on our hypothesize that proteins involved in osteoclast differentiation may serve as a suitable source of non-OPs, we extract 488 peptides from such proteins, forming the set ${\mathbb{N}}^{h}=\{P_{\text{1}}^{{\mathbb{N}}^{h}}\text{,}\ldots P_{\text{488}}^{{\mathbb{N}}^{h}}\}$.

Further details regarding our hypothesis for selecting non-OPs are provided below. This hypothesis is based on the distinct functional roles that proteins play in the bone remodeling process. Specifically, proteins involved in osteoclast differentiation contribute to bone breakdown, whereas those with osteogenic activity, such as bone morphogenetic proteins, promote bone formation by supporting osteoblast differentiation. Due to this functional divergence, proteins that promote osteoclast activity are unlikely to simultaneously support osteogenic functions. To do this, we select the proteins O88942, Q9CWT3, and Q5T9C2 from UniProt [24] as the source for generating non-OPs. To extract non-OPs from these proteins, PeptideCutter [25] is utilized to predict the potential cleavage sites in these proteins by chymotrypsin and trypsin enzymes.

### 2.3. SimPep framework

**Fig 1:**
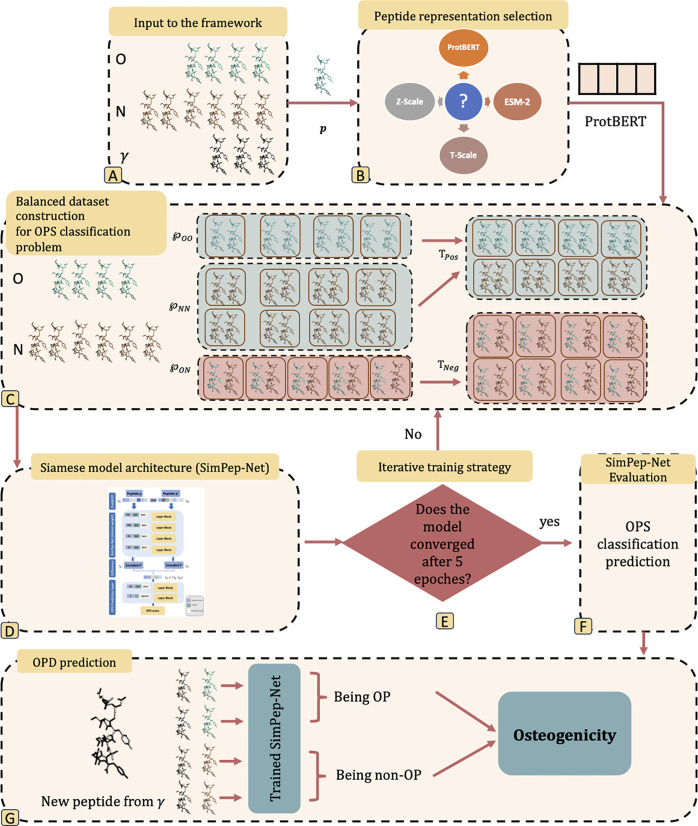
Overview of the SimPep framework, a seven-step process for osteogenic peptide detection (OPD). **(A)** Input sets: O (positive training set), N (negative training set) and γ (test set), **(B)** Peptide representation based on biologically features (Z-scale and T-scale) and embeddings obtained from protein language models (ProtBERT and ESM-2); ProtBERT is selected as the optimal representation, **(C)** Balanced dataset construction ($T_{pos}\cup T_{neg}$) for osteogenic peptide similarity (OPS) classification problem where ${℘}_{OO}$: the pairs of known OPs share the same osteogenic properties, ${℘}_{NN}$: the pairs of non-OPs also share the same osteogenic properties, ${℘}_{ON}$: the pairs of OPs and non-OPs exhibit varying osteogenicity; $T_{pos}={℘}_{OO}\cup {{℘}^{\prime }}_{NN},\;{{℘}^{\prime }}_{NN}\;\subseteq {℘}_{NN},\;\left|{{℘}^{\prime }}_{NN}\right|=\left|{℘}_{\text{OO}}\right|$ and $T_{neg}$ = randomly oversampled ${℘}_{ON}$ where $\left|T_{neg}\right|=|T_{pos}|$, **(D)** SimPep-Net: a siamese model architecture for OPS classification prediction, **(E)** Iterative training: if accuracy is unstable after 5 epochs, a new balanced dataset is generated (repeat C) for retraining, **(F)** SimPep-Net evaluation for OPS classification prediction, **(G)** OPD prediction: unknown peptides in γ are paired with known peptides to infer osteogenicity using SimPep-Net.

Due to the limited number of available OPs and non-OPs, training a traditional DL model for OPD prediction is not feasible. To overcome this limitation, we introduce SimPep, a seven-step framework (see Fig 1) designed for OPD prediction using a DL-based approach. In this framework, the OPD problem is reformulated as the OPS classification problem, where a DL model named SimPep-Net is designed to train for distinguishing between similar and dissimilar peptide pairs based on their osteogenic properties. After training, SimPep-Net model is used for OPD prediction by pairing each unseen peptide in the test set with all peptides in the training set. The model predicts the similarity for each pair, and then a similarity-aggregated scoring function is applied to determine whether each peptide in the test set is an OP or a non-OP. The key steps of the SimPep framework are as follows:

1. Input to the framework.
2. Peptide representation selection.
3. Balanced dataset construction for OPS classification problem.
4. Siamese model architecture (SimPep-Net) for OPS classification prediction.
5. Iterative training strategy for enhancing SimPep-Net generalization.
6. SimPep-Net evaluation for OPS classification prediction.
7. OPD prediction.

The details of each step are explained in the following sub-sections.

#### 2.3.1. Input to the framework.

As the input to the SimPep framework, three distinct peptide sets are identified: a positive training, a negative training and a test sets. These sets form the foundation for constructing datasets used to train and evaluate the DL model:

- Positive training set: $O=\{P_{1}^{O},\ldots P_{n_{1}}^{O}\}$, where $|O|=n_{1}\;$, O⊆𝕆 and set 𝕆 includes 108 peptides extracted from OP-AND database.
- Negative training set: $N=\{P_{1}^{N},\ldots P_{n_{2}}^{N}\}$, where $|N|=n_{2}$, N⊆N and set ℕ corresponds to either $N={\mathbb{N}}^{r}$ (300 peptides from random proteins) or $N={\mathbb{N}}^{h}$ (488 peptides from proteins involved in osteoclast differentiation).
- The test peptide set: $\gamma =\left\{P_{1}^{\gamma },\ldots P_{n_{3}}^{\gamma }\right\}$, where $|\gamma |=n_{3}\;$ and γ∩(O∪N)=∅.

#### 2.3.2. Peptide representation selection.

One of the most critical aspects of the DL models is how the input data is represented, as effective encoding directly impacts the model’s ability to accurately classify samples. In the context of OPD, the choice of encoding strategy for peptide sequences plays a vital role in distinguishing between OPs and non-OPs. In this study, we employ two distinct encoding strategies: biologically derived features and embeddings obtained from protein language models.

For biologically inspired descriptors, we utilize Z-scale [29] and T-scale [30] representations. Both methods encode each amino acid using five physicochemical properties, resulting in a matrix of size l×5 for a peptide of length l. The Z-scale [29] represents amino acids based on lipophilicity, steric bulk, polarity, electronic effects, and miscellaneous properties. The T-scale [30], derived through multidimensional scaling of a broad range of physicochemical characteristics, is optimized for peptide classification and quantitative structure–activity relationship (QSAR) analysis. To generate a fixed-length vector suitable for model input, each peptide matrix is averaged across all amino acids, yielding a final vector representation $v_{P}$. To extract Z-scale or T-scale, the Peptides R package [31] is utilized to compute amino acid descriptors per sequence.

In parallel, we explore transformer-based language models trained on protein sequences, which treat peptides as sequences of amino acids analogous to sentences in natural language. In this analogy, amino acids act as words, and transformer-based masked language models learn contextualized representations of these sequences. We evaluate two state-of-the-art models: ProtBERT [32] (1024-dimensional) and ESM-2 [33] (1280-dimensional). Each peptide sequence is tokenized and passed through the respective model to extract the last hidden layer representations. These token-level embeddings are then averaged, excluding special tokens, to produce a fixed-length vector $v_{P}$ for each peptide.

Among these models, ProtBERT [32], built on the BERT architecture and trained on the UniRef100 dataset, has demonstrated strong performance (see 3.4. Assessment of peptide representation on the performance of the SimPep framework section) in capturing structural and functional information embedded in protein sequences. Prior studies have shown that ProtBERT [32] embeddings encode meaningful biochemical and functional attributes, making them well-suited for downstream peptide classification tasks [34].

#### 2.3.3. Balanced dataset construction for OPS classification problem.

Due to the limited number of available OPs and non-OPs, training a traditional DL model for OPD prediction is not feasible. To overcome this limitation, we reformulate the OPD task as OPS classification problem, where the model learns to distinguish between similar and dissimilar peptide pairs based on their osteogenic properties.

To this end, we construct a large training dataset by generating peptide pairs from two sets: O (OPs/positives) and N (non-OPs/negatives). Each pair is assigned a similarity label as follows:

- Label 1: similar peptide pair (OP–OP or non-OP–non-OP)
- Label 0: dissimilar peptide pair (OP–non-OP or non-OP-OP)

However, this pairing approach introduces a data imbalance due to the scarcity of OPs relative to non-OPs. Specifically:

- Most label-1 pairs are non-OP–non-OP, which may bias the model toward learning non-osteogenic similarity.
- The number of label-0 (OP–non-OP) pairs is often smaller than label-1 pairs, exacerbating class imbalance.

To address this issue, we introduce a balancing function named Cons−Train, which constructs a sufficiently large and well-balanced training dataset that captures meaningful osteogenic similarity. This function takes the initial positive (O) and negative (N) training peptide sets as input and generates positive and negative training datasets for OPS classification problem: $T_{pos}$ as the positive paired peptide training dataset (label = 1) and $T_{neg}$ as the negative paired training dataset (label = 0). The main steps of Cons−Trainare as follows (see Fig 1C):

1 Construct same-class pairs:
  -  OP–OP pairs with similar osteogenic properties: ${℘}_{OO}=\{<P,P^{\prime }>|\;P,P^{\prime }\in O\}$,
  -  non-OP–non-OP pairs (also labeled as similar): ${℘}_{NN}=\{<P,P^{\prime }>|\;P,P^{\prime }\in N\}$.
2 Construct OP–non-OP pairs: ${℘}_{ON}=\left\{<P,P^{\prime }>\right|P\in O,\;P^{\prime }\in N\}$.
3 Balance the similar class: since $\left|{℘}_{NN}\right|>|{℘}_{OO}|$, randomly select a ${{℘}^{\prime }}_{NN}\subset {℘}_{NN}$ such that $\left|{{℘}^{\prime }}_{NN}|=|{℘}_{OO}\right|$.
4 Form the positive paired peptide training dataset: $T_{pos}={℘}_{OO}\cup {{℘}^{\prime }}_{NN}$ where ${|T}_{pos}|=2|{℘}_{OO}|$.
5 Balance the dissimilar class: if $\left|{℘}_{ON}\right|<\left|T_{pos}\right|$ incorporating random oversampling on ${℘}_{ON}$ to form $T_{neg}$ such that ${|T}_{pos}\left|={|T}_{neg}\right|$.
6 Label assignment:
  -  For each $<P,P^{\prime }>\in T_{pos}$: assign label $y_{<P,P^{\prime }>}=1$,
  -  For each $<P,P^{\prime }>\in T_{neg}$: assign label $y_{<P,P^{\prime }>}=0$.
7 Output: return $T_{pos}$ and $T_{neg}\;$as the balanced positive and negative paired training datasets, respectively.

#### 2.3.4. Siamese model architecture (SimPep-Net) for OPS classification prediction.

**Fig 2:**
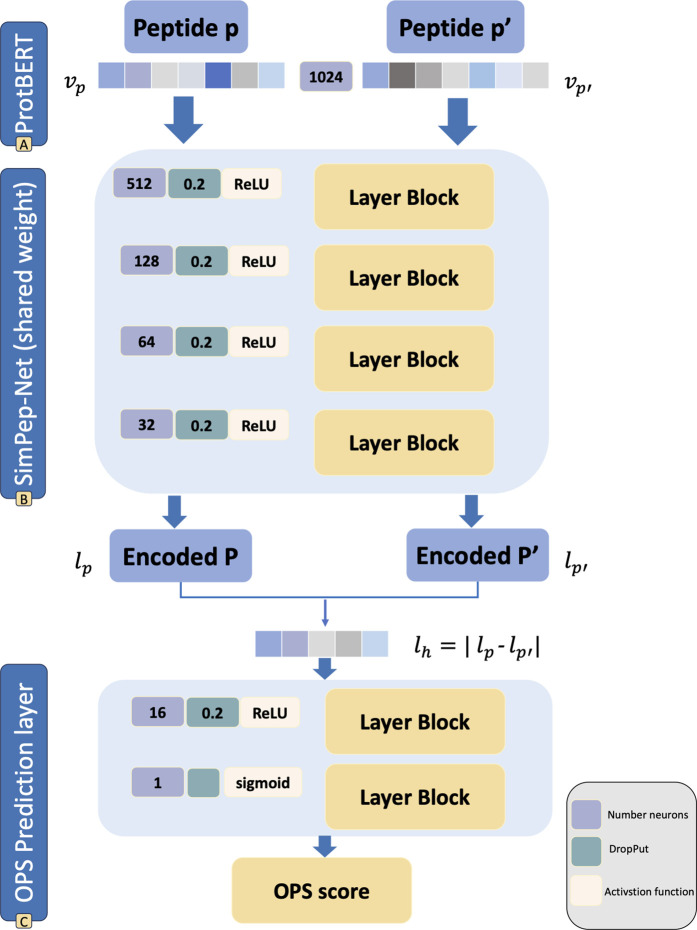
The architecture of SimPep-Net model. **(A)** A pair of peptides $<P,P^{\prime }>$ is provided as input to SimPep-Net, with each peptide encoded to a 1024-dimensional vector using the pre-trained ProtBERT model ($v_{P}$ and $v_{P^{\prime }})$, **(B)** Each vector is mapped individually to a 32-dimensional ($l_{P}$ and $l_{P^{\prime }})$ latent space via a non-linear function $f:R^{1024}\to R^{32}$, **(C)** The absolute difference between the two latent vectors is computed and passed through a fully connected layer with 16 neurons followed by a sigmoid activation to predict peptide similarity.

In the fourth step, we design an SNN architecture called SimPep-Net, which processes paired peptides to learn the OPS task by distinguishing between similar and dissimilar pairs. SimPep-Net comprises two identical channels to embed the input peptides into a shared latent space, ensuring that peptides with the same osteogenic properties are positioned closer together. The network is trained so that if both peptides in a given pair exhibit similar osteogenic properties, SimPep-Net outputs a value of one; otherwise, the output is zero, based on the predicted probability. Fig 2 illustrates the proposed SimPep-Net architecture.

In particular, for each pair of peptides, $<P,P^{\prime }>\in T_{pos}\cup T_{neg}$, the peptides are processed separately (see Fig 2A). The ProtBERT-based representations of peptides P and $P^{\prime }$, $v_{P}$ and $v_{P^{\prime }}$, respectively, pass through dedicated channels. Within the SimPep-Net architecture, these channels learn a non-linear function where $f(v_{P})=l_{P}$ and $f(v_{P^{\prime }})=l_{P^{\prime }}$. The resulting vectors $l_{P}$ and $l_{P^{\prime }}$ show their embeddings in the latent space (see Fig 2B). The architecture of function f encompasses four dense hidden layers comprising 512, 128, 64, and 32 neurons. These layers reduce the input vectors of $v_{p}$ and $v_{p^{\prime }}$ with length of 1024 to $l_{p}$ and $l_{p^{\prime }}$, respectively, with length of 32. To introduce non-linearity into the data, rectified linear unit (ReLU) serves as the activation function in these layers. Furthermore, to prevent overfitting, a dropout of 0.2 is applied after each layer.

The primary objective of the SimPep-Net model is to facilitate the proximity of peptides with similar osteogenic properties. To achieve this, an elementwise subtraction operation is performed between the vectors $l_{P}$ and $l_{P^{\prime }}$, generating a new vector named $l_{h}=|l_{P}-l_{P^{\prime }}|$. The resulting vector, $l_{h}$, encapsulates information about the osteogenicity similarity of the given peptide pair (see Fig 2C). To investigate this relationship further, $l_{h}$ undergoes processing through one dense hidden layer comprising 16 neurons, respectively. This layer is activated using ReLU and implements a dropout of 0.2 to mitigate overfitting. The final layer comprises a single neuron with a sigmoid activation function (${\overset{―}{y}}_{<P,P^{\prime }>}$), distinguishing whether the given pair of peptides exhibits similar osteogenic properties or not. In other words, it detects whether the given pair of peptides are both osteogenic, non-osteogenic, or even one is osteogenic and the other is not. Considering the sigmoid function’s output, representing the likelihood of peptides P and $P^{\prime }$ sharing the same osteogenicity within the range [0,1], probabilities exceeding 0.5 are interpreted as identical properties, while those below 0.5 imply differing osteogenic properties. However, to mitigate disparities between the predicted and actual outcomes, we employ the binary cross-entropy loss function (L) for each $<P,P^{\prime }>$ pair, compiling the SimPep-Net model that is calculated as follows:


$$ℒ\left(y_{<P,P^{\prime }>},{\overset{―}{y}}_{<P,P^{\prime }>}\right)=-\left[y_{<P,P^{\prime }>}\times log\left({\overset{―}{y}}_{<P,P^{\prime }>}\right)+\left(1-y_{<P,P^{\prime }>}\right)\times log\left(1-{\overset{―}{y}}_{<P,P^{\prime }>}\right)\right],$$

where, $y_{<P,P^{\prime }>}$ indicates the actual state of osteogenicity between peptides P and $P^{\prime }$ and ${\overset{―}{y}}_{<P,P^{\prime }>}$ shows the predicted state by the SimPep-Net.

#### 2.3.5. Iterative training strategy for enhancing SimPep-Net generalization.

To train SimPep-Net, we use the Cons−Train(O,N) function to construct a balanced paired peptide training dataset. In steps three and five of this function, samples are randomly selected to ensure class balance, introducing variability into each generated dataset. To improve the model’s generalization and robustness, we adopt an iterative training strategy as described below.

Initially, positive and negative balanced paired peptide training datasets are generated using Cons−Train(O,N), resulting in sets $T_{pos}$ and $T_{neg}$, respectively, which are then used to train SimPep-Net. If the training accuracy does not stabilize after 5 epochs, we regenerate a new balanced paired peptide training dataset using Cons−Train(O,N) and resume training. Importantly, the model is re-initialized with the weights learned in the previous iteration, allowing it to train previously acquired knowledge, continuing to refine its performance on a new dataset. The inherent randomness in the sample selection steps of Cons−Train ensures that each training set is diverse. This diversity helps to prevent overfitting and significantly improves the model’s ability to generalize to unseen peptide pairs.

#### 2.3.6. SimPep-Net evaluation for OPS classification prediction.

SimPep-Net is trained to perform the OPS classification task. To rigorously evaluate its performance, we construct a dedicated test set using the Sim−Test(γ,L) function. This function accepts two inputs: the test peptide set γ and a corresponding label vector L, where each $L_{P}\in \{0,1\}$ indicates the ground-truth identity of peptide P in γ ($L_{P}=1$ for OP and $L_{P}=0$ for non-OP). Sim−Test enables a systematic assessment of the model’s ability to distinguish between similar (OP–OP or non-OP–non-OP) and dissimilar (OP–non-OP or non-OP-OP) peptide pairs, based on their osteogenic properties. Crucially, none of the peptides in γ are included in the training process, making this evaluation a strict generalization test and offering an unbiased measure of the model’s performance. The Sim−Test(γ,L function proceeds through the following steps:

1 Generate all valid peptide pairs: $\rho =\left\{<P,P^{\prime }>\right|\;P\text{,}P^{\prime }\in \gamma \;\;P\neq P^{\prime }\}$.
2 Assign true labels to each pair: each pair $<P,P^{\prime }>\;$ is assigned a binary label $y_{<P,P^{\prime }>}$ based on the ground-truth labels of the individual peptides:

$$\begin{array}{c} \;y_{<P,P^{\prime }>}=\left\{ \begin{array}{c} @l1\;L_{P}=L_{P^{\prime }}\;(\text{OP}-\text{OP or non}-\text{OP}-\text{non}-\text{OP})\;\\ 0\;L_{P}\neq L_{P^{\prime }}\;(\text{OP}-\text{non}-\text{OP or non}-\text{OP}-\text{OP})\; \end{array}\right.\; \end{array}$$

3 Predict similarity using SimPep-Net: ${\overset{―}{y}}_{<P\text{,}P^{\prime }>}=SimPep-Net(P,P^{\prime })$.
4 Threshold prediction:

$$\begin{array}{c} {\hat{y}}_{<P\;P\prime >}=\left\{ \;\begin{array}{c} @l1\;{\overset{―}{y}}_{<P\;P^{\prime }>}\geq 0.5\\ 0\;otherwise\text{\textbackslash{} \textbackslash{} } \end{array}\right.\; \end{array}$$

1. Evaluate prediction accuracy: the predicted labels ${\hat{y}}_{<P,P^{\prime }>}$ are compared against the true labels $y_{<P,P^{\prime }>}$ to calculate performance metrics such as accuracy, specificity, and sensitivity for the OPS classification task.

#### 2.3.7. OPD prediction.

The last step of SimPep framework outlines how to use trained SimPep-Net for OPD prediction. We introduce a similarity-aggregated scoring function called OP−Pre(γ,O,N), which takes three parameters: the test peptide set (γ), the positive training set (O), and the negative training set (N). This function predicts whether the peptides in γ are OP or non-OP. The key steps of this function are as follows:

1. Pairing with known peptides in the training set: $\begin{array}{c} \forall \text{\textbackslash{} }P\in \gamma \;X_{O}^{P}=\left\{<P\;P_{1}^{O}>\ldots <P\;P_{\left|O\right|}^{O}>\right\}\;X_{N}^{P}=\left\{<P\;P_{1}^{N}>\ldots <P\;P_{\left|N\right|}^{N}>\right\} \end{array}$
2. Similarity prediction: $\begin{array}{c} \forall \text{\textbackslash{} }<P\;P\prime >\in X_{O}^{P}\cup X_{N}^{P}\;{\overset{―}{y}}_{<P\;P^{\prime }>}=SimPep-Net\left(P\;P^{\prime }\right) \end{array}$
3. Score aggregation: $\;{\mathbb{C}}_{O}=\sum_{<P,P^{\prime }>\in X_{O}^{P}}{\overset{―}{y}}_{<P,P^{\prime }>},\;{\mathbb{C}}_{N}=\sum_{<P,P^{\prime }>\in X_{N}^{P}}{\overset{―}{y}}_{<P,P^{\prime }>}$.
4. Osteogenic probability calculation: ${\;\;\zeta }_{ost}=(1-\frac{{\mathbb{C}}_{N}}{|N|})+\frac{{\mathbb{C}}_{O}}{|O|}$.
5. Output: return $\varrho_{P}=\frac{\zeta_{ost}}{2}$ for each P∈γ, representing the predicted likelihood that P is an OP.

## 3. Results and discussion

The assessment of the SimPep framework’s performance involves several experiments based on specific evaluation criteria. This section initially introduces the evaluation criteria and the statistics of the applied dataset. The framework utilizes five-fold cross-validation for training and testing, followed by parameter tuning and evaluation of peptide representation generation, ultimately choosing ProtBERT [32] as the preferred approach. This selection is based on ProtBERT’s ability to generate rich, contextualized embeddings for peptide sequences [34].

To demonstrate the effectiveness of the SimPep framework in solving the OPD prediction, a five-fold cross-validation is performed using OPs from the OP-AND database and non-OPs from osteoclast proteins. The hypothesis that non-OPs from osteoclast proteins serve as an appropriate negative OP set is validated through another five-fold cross-validation, comparing these non-OPs with random peptides from a previous study [22]. Furthermore, the performance of the SimPep framework for predicting the osteogenic potential of peptides is benchmarked against three baseline machine learning methods: random forest (RF), support vector machine (SVM), and XGBoost.

Next, the framework is evaluated on two external test peptide pools. The first external peptide pool is defined with OPs published between 2022 and 2024. The second one is experimentally validated non-OPs from earlier research [22].

As a case study, the SimPep framework is applied to identify potential OPs in casein proteins. A pipeline is also proposed to prioritize potential OPs predicted by SimPep for further experimental testing. Through these steps, a peptide derived from camel milk alpha s1-casein is recommended for experimental investigation due to its potential osteogenic properties.

### 3.1. Evaluation criteria

To evaluate the performance of the SimPep-Net model and SimPep framework for OPS classification and OPD prediction, respectively, three main criteria are applied:

- Accuracy (ACC), a fundamental metric in assessing model performance, quantifies the extent to which the model accurately predicts both positive and negative outcomes. It is calculated as follows:

$$ACC=\frac{TP+TN}{TP+TN+FP+FN},$$

where, TP and TN indicate true positive and true negative, respectively, as well as FP and FN abbreviate false positive and false negative.

- Specificity (SPC) score calculates the ability of the model in the identification of negative samples, which is expressed mathematically as below:

$$SPC=\frac{TN}{TN+FP}.$$

- Sensitivity (SEN) score measures the power of the model in correctly predicting positive samples and is formulated as:

$$SEN=\frac{TP}{TP+FN}.$$

### 3.2. Five-fold cross-validation approach to make train and test sets

To evaluate SimPep-Net model and SimPep framework for OPS and OPD predictions, respectively, we use five-fold cross-validation. Here we define the approach for five-fold cross-validation on the OP set $\begin{array}{c} O=\left\{P_{\text{1}}^{O}\text{,}\ldots P_{n_{O}}^{O}\right\}\text{\textbackslash{} } \end{array}$ and non-OP set $\mathbb{N}=\{P_{1}^{\mathbb{N}},\ldots P_{n_{\mathbb{N}}}^{\mathbb{N}}\}$, which are randomly split into five equal-sized, disjoint subsets as follows:

- ${⋃}_{j=1}^{5}{\text{fold}}_{j}^{𝕆}=𝕆,\;{⋂}_{j=1}^{5}{\text{fold}}_{j}^{𝕆}=\emptyset$ and $\left|{\text{fold}}_{j}^{𝕆}|=|{\text{fold}}_{k}^{𝕆}\right|$for all j≠k,
- ${⋃}_{j=1}^{5}{\text{fold}}_{j}^{\mathbb{N}}=\mathbb{N},\;{⋂}_{j=1}^{5}{\text{fold}}_{j}^{\mathbb{N}}=\emptyset$and $\left|{\text{fold}}_{j}^{\mathbb{N}}\right|=\left|{\text{fold}}_{k}^{\mathbb{N}}\right|$for all j≠k,

where |.| shows the size of the set.

In each iteration i∈{1,…,5} of five-fold cross-validation, test and train peptide sets are constructed as follows:

- Test peptide set $\gamma_{i}$ is constructed based on the *i*^th^ folds of both OP and non-OP sets as follows:

$\begin{array}{c} \gamma_{i}={\text{fold}}_{i}^{𝕆}\cup {\text{fold}}_{i}^{\mathbb{N}} \end{array}$.

For each peptide $P\in \gamma_{i}$, the corresponding element in the label vector L denoted as $L_{P}$ indicates the true class label as follows:


$$\begin{array}{c} L_{p}=\{1\;\;\;{P\in fold}_{i}^{O}\;0\;{\;P\in fold}_{i}^{N}\; \end{array}$$

- For each iteration i∈{1,2,…,5}, the positive and negative training sets are constructed by combining all folds except the *i*^th^ fold from the positive and negative data as follows:

$$O_{i}={⋃}_{j=1,j\neq i\;}^{5}{fold}_{j}^{\text{O}},\;N_{i}={⋃}_{j=1,j\neq i}^{5}{fold}_{j}^{\text{N}}$$

### 3.3. Hyperparameter tuning

To further optimize the performance of the SimPep-Net model, we systematically tune key hyperparameters, including the dropout, learning rate, and optimizer. These experiments are defined to ensure that the model is neither overfitting nor underfitting and generalizes well to unseen peptide sequences. This is achieved through a five-fold cross-validation process, creating distinct training and testing sets ($O_{i},\;N_{i\;}$, and $\gamma_{i}$) in each iteration, based on OP and non-OP sets (𝕆,$\;{\mathbb{N}}^{h}$).

#### 3.3.1. Dropout tuning.

**Fig 3:**
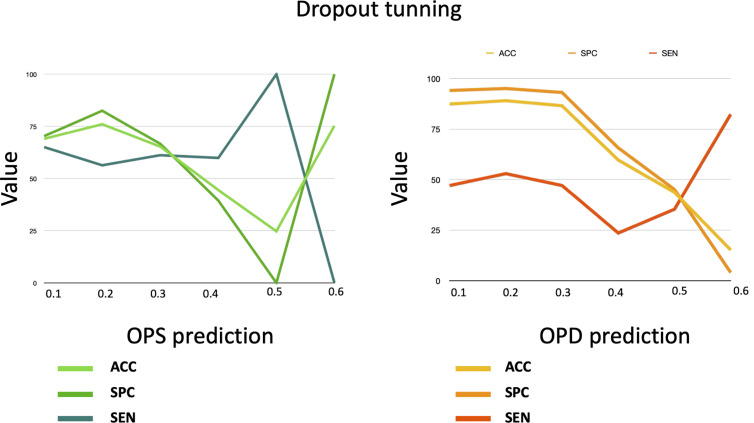
The validation of the SimPep-Net model for OPS prediction and the SimPep framework for OPD prediction under different dropout settings.

We tune the dropout rate by testing values between 0.1 and 0.6 for each i∈{1,…,5} of five-fold cross-validation, where the SimPep-Net model is trained on positive ($O_{i}$) and negative ($N_{i}$) datasets and tested on i^th^ fold ($\gamma_{i}$). For each dropout setting, Fig 3 shows the average performance of five-fold cross-validation on both the OPS and the OPD prediction tasks.

According to the results, a dropout rate of 0.2 provides the best trade-off between accuracy, specificity, and sensitivity across both tasks. Higher dropout rates (≥ 0.4) cause significant performance deterioration due to over-regularization, while lower dropout rates (< 0.2) do not improve generalization.

#### 3.3.2. Learning rate tuning.

**Fig 4:**
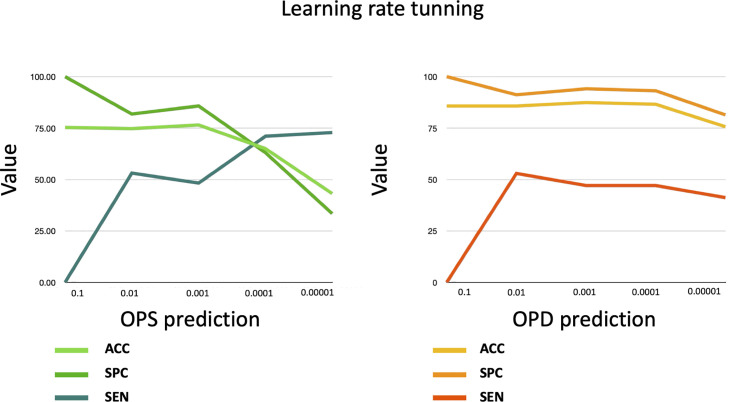
The validation of the SimPep-Net model for OPS prediction and the SimPep framework for OPD prediction under different learning rate settings.

For each learning rate α∈{0.1,0.01,..,0.00001}, the SimPep-Net model using five different training dataset ($O_{i}$ and $N_{i}$) and validated using a five-fold cross-validation approach. Each of the five folds ($\gamma_{i}$) serves as a test set. The average performance values five-fold cross-validation in both the OPS and the OPD prediction (see Fig 4) tasks present that while very large learning rates (α = 0.1) cause unstable behavior and poor convergence, very small learning rates (α ≤ 0.0001) lead to slow learning and suboptimal performance. Therefore, a learning rate of α = 0.001 provides the best balance between accuracy and generalization across both tasks. As a result, α = 0.001 is selected as the final learning rate for SimPep-Net.

#### 3.3.3. Optimizer selection.

**Fig 5:**
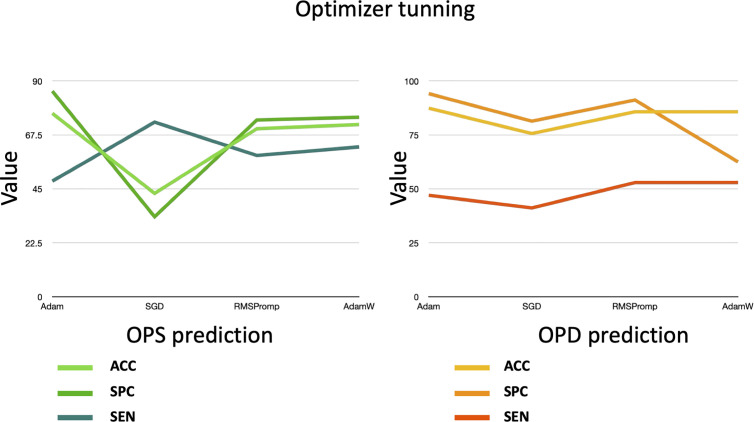
The validation of the SimPep-Net model for OPS prediction and the SimPep framework for OPD prediction under different optimizer configurations.

Finally, we evaluate different optimizers to assess their impact on SimPep-Net performance. We assess Adam, SGD, RMSprop, and AdamW as the optimizers using the previously selected learning rate (α = 0.001) and dropout rate (0.2). The results are illustrated in Fig 5 for both OPS and OPD prediction tasks.

According to the results, Adam optimizer consistently outperforms the other optimizers, achieving the highest accuracy, a better trade-off between specificity and sensitivity, and more stable training behavior, for both OPS and OPD prediction tasks. Based on this analysis, Adam is selected as the final optimizer for SimPep-Net.

### 3.4. Assessment of peptide representation on the performance of the SimPep framework

In this sub-section, we assess our framework into two distinct representation strategies for the peptide sequence: biologically derived features (Z-scale [29] and T-scale [30]) and embeddings obtained from protein language models (ProtBERT and ESM-2) for OPS and OPD prediction tasks. The evaluation uses a five-fold cross-validation on an OP (𝕆) and non-OP (${\mathbb{N}}^{h}$) set, iterated five times (i = 1 to 5). In each iteration, the framework receives positive ($O_{i}$), negative ($N_{i}$) training sets and test set ($\gamma_{i}$).

Table 1 and Table 2 represent the performance of each peptide representation for OPS and OPD prediction tasks, respectively, based on the average of five-fold cross-validation.

**Table 1:** The validation of the SimPep-Net model for the OPS classification problem based on peptide representation by ProtBERT and ESM-2 embeddings, and Z-Scale and T-Scale biological features.

Peptide representation	ACC (%)	SPC (%)	SEN (%)
ProtBERT	76.06	66.30	80.25
ESM-2	66.47	57.97	80.52
Z-scale	63.66	49.07	74.13
T-scale	66.41	49.13	73.74

**Table 2:** The validation of the SimPep framework for OPD based on peptide representation by ProtBERT and ESM-2 embeddings, and Z-Scale and T-Scale biological features.

Peptide representation	ACC (%)	SPC (%)	SEN (%)
ProtBERT	86.87	92.86	59.77
ESM-2	86.84	93.80	52.01
Z-scale	79.92	87.75	44.36
T-scale	78.90	87.12	41.51

The results clearly show that ProtBERT consistently outperforms the other representations in both the OPS and OPD prediction tasks. While Z-scale and T-scale capture important physicochemical properties (e.g., hydrophilicity, size and charge), their aggregated descriptors do not fully capture sequence context, limiting their effectiveness on this task. Based on this comprehensive comparison, we select ProtBERT as the core embedding model for the SimPep framework.

### 3.5. Evaluating SimPep

In the previous sub-section, we selected ProtBERT as an appropriate approach for peptide representation. The first row of Tables 1 and 2 shows that the average performance of the SimPep-Net model and the SimPep framework on OPS and OPD prediction tasks across the five folds. The training and test sets are defined on the OP set $𝕆=\{P_{1}^{𝕆},\ldots ,P_{108}^{𝕆}\}$ and the non-OP set ${\mathbb{N}}^{h}=\left\{P_{\text{1}}^{{\mathbb{N}}^{h}}\text{,}\ldots P_{\text{488}}^{{\mathbb{N}}^{h}}\right\}$. For each of the five iterations (i = 1 to 5), the SimPep framework is run with three input sets: $O_{i}$,${\;N}_{i}$ and $\gamma_{i}$. After training the framework on $O_{i}$ and ${\;N}_{i}$, the results of the evaluation of OPS and OPD predictions on $\gamma_{i}$ are available in Tables 3 and 4, respectively.

**Table 3:** The SimPep-Net performance for the OPS classification problem in each fold of five-fold cross-validation.

Fold	ACC (%)	AUC-ROC (%)	SPC (%)	SEN (%)
$\gamma_{1}$	76.76	76.40	79.56	75.54
$\gamma_{2}$	85.54	86.39	58.67	97.39
$\gamma_{3}$	71.31	71.93	54.28	78.44
$\gamma_{4}$	74.09	70.59	73.50	74.34
$\gamma_{5}$	72.62	70.93	65.51	75.57
AVG	76.06	75.25	66.30	80.25
STD	5.67	6.64	1.03	9.69

**Table 4:** The SimPep performance in OPD prediction in each fold of five-fold cross-validation.

Fold	ACC (%)	AUC-ROC (%)	SPC (%)	SEN (%)
$\gamma_{1}$	89.11	76.84	92.00	76.35
$\gamma_{2}$	90.76	88.89	99.53	52.50
$\gamma_{3}$	85.42	72.70	92.01	55.00
$\gamma_{4}$	83.64	75.36	88.76	59.99
$\gamma_{5}$	85.42	70.64	92.01	54.99
AVG	86.87	76.88	92.86	59.77
STD	2.94	7.12	3.98	9.66

For OPS prediction, the SimPep-Net model achieves an average accuracy of 76.06%±5.67, an AUC-ROC of 77.25%±6.64, a specificity of 66.30%±1.03, and a sensitivity of 80.26%±9.69 across the five-fold cross-validation. We consider AUC-ROC as a supporting metric to prioritize models during training and selection. Given the class imbalance in our dataset, AUC-ROC provides a more stable indicator of overall model discrimination capability by capturing the trade-off between true positive and false positive rates, regardless of threshold.

These results highlight the model’s robustness in accurately predicting the osteogenic property of previously unseen peptide pairs, demonstrating its ability to generalize beyond the training data.

The performance of the framework is evaluated by calculating key metrics across relevant evaluation criteria. According to Table 4, the SimPep framework achieves an average accuracy of 86.87%±2.94, an AUC-ROC of 76.88%±7.12, a specificity of 92.86%±3.98, and a sensitivity of 59.77%±9.66 for OPD prediction, across five-fold cross-validation. The sensitivity score, which measures the model’s ability to accurately identify true OPs, is particularly significant given the imbalance in the test set, which consists of 17 OPs and 130 non-OPs. Despite this disparity, a sensitivity of 60% indicates that the model is reliable in recognizing OPs. This suggests that the predicted OPs are likely to exhibit osteogenic potential.

### 3.6. Hypothesis validation on non-OPs

This sub-section assesses the hypothesis that peptides derived from proteins involved in osteoclast differentiation (${\mathbb{N}}^{h},\;\left|{\mathbb{N}}^{h}\right|=488$) can be used as a reliable set of non-OPs. To evaluate this, the study compares the performance of the framework in OPS and OPD prediction tasks, using $N^{h}$ as the non-OP set and a randomly selected set of proteins (${\mathbb{N}}^{r},\;\;\left|{\mathbb{N}}^{r}\right|=300$) with no known osteogenic involvement as a negative training set. This comparison allows us to evaluate if the peptides from osteoclast differentiation proteins (${\mathbb{N}}^{h}$) accurately reflect non-OP behavior in the framework. Five-fold cross-validation is applied to two sets of peptides: $𝕆=\{P_{1}^{𝕆},\ldots ,P_{108}^{𝕆}\}$ and ${\mathbb{N}}^{r}=\left\{P_{\text{1}}^{{\mathbb{N}}^{r}}\text{,}\ldots P_{\text{300}}^{{\mathbb{N}}^{r}}\right\}$, representing OPs and non-OPs, respectively. For each of five iterations, i=1…5, the SimPep framework is used with the three sets: $O_{i}$, $N_{i}$ and $\gamma_{i}$ as inputs.

When comparing the obtained results in Tables 3 and 5, the performance of SimPep-Net trained on the hypothesized negative peptide pool significantly outperforms the model trained on the random negative pool, showing approximately an 11% improvement in accuracy and a 13% increase in sensitivity. The sensitivity score is particularly important as it highlights the model’s ability to correctly identify true positives, which is critical for ensuring the reliability of predicted osteogenic peptides, especially when their numbers are limited.

**Table 5:** The SimPep-Net performance using ${\mathbb{N}}^{r}$as the non-OP set for the OPS classification problem.

Fold	ACC (%)	SPC (%)	SEN (%)
$\gamma_{1}$	66.51	66.69	66.40
$\gamma_{2}$	59.49	81.34	44.69
$\gamma_{3}$	67.41	54.84	75.63
$\gamma_{4}$	64.91	58.78	68.92
$\gamma_{5}$	69.19	49.26	82.23
AVG	65.50	62.18	67.57
STD	3.69	12.44	14.20

Comparing OPD prediction using our hypothesis non-OP set ${\mathbb{N}}^{h}$ (see Table 4) and the random non-OP set ${\mathbb{N}}^{r}$(see Table 6) shows that employing ${\mathbb{N}}^{h}$ as non-OP set outperforms ${\mathbb{N}}^{r}$ in terms of accuracy and specificity with an improvement of approximately 8% and 5%, respectively.

**Table 6:** The SimPep framework performance using ${\mathbb{N}}^{r}$as non-OP set for OPD prediction.

Fold	ACC (%)	SPC (%)	SEN (%)
$\gamma_{1}$	80.0	87.93	59.09
$\gamma_{2}$	78.75	81.03	72.72
$\gamma_{3}$	81.01	91.37	52.38
$\gamma_{4}$	78.481	86.20	57.14
$\gamma_{5}$	81.01	93.10	47.61
AVG	79.85	87.92	57.79
STD	1.20	4.72	9.45

The size of each negative peptide fold constructed based on ${\mathbb{N}}^{h}$ is larger than each negative peptide fold constructed based on ${\mathbb{N}}^{r}$ since $\left|{\mathbb{N}}^{h}\right|=488>\left|{\mathbb{N}}^{r}\right|=300$. It may influence the obtained evaluation scores.

To interpret the results, we employ the confidence interval (CI) as a statistical criterion. The CI criterion provides an interval within which the true population parameter is expected to lie, given a specified level of confidence, by computing a lower and upper bound around the estimation. The CI for each fold i is calculated as follows:


$$CI=AVG\;\pm z*(STD/\sqrt{|\gamma_{i}|})$$

where $|\gamma_{i}|$ shows the number of samples in the i^th^ fold, and the value of z represents the distance measured in standard deviations from the mean in a normal distribution. For a 95% confidence level, z is 1.96. A smaller CI indicates a more precise estimate, while a larger CI suggests greater uncertainty. The CI scores are calculated based on two different non-OPs sets, ${\mathbb{N}}^{h}$ and ${\mathbb{N}}^{r}$, for each evaluation criterion, which is presented in Table 7.

**Table 7:** CI criterion for comparing the performance of the framework for two different non-OP sets.

Evaluation	non-OP set	CI		
		ACC (%)	SPC (%)	SEN (%)
**OPS prediction**	${\mathbb{N}}^{h}$	4.97	9.01	8.50
	${\mathbb{N}}^{r}$	3.24	10.91	12.45
**OPD prediction**	${\mathbb{N}}^{h}$	2.58	3.49	8.47
	${\mathbb{N}}^{r}$	1.05	4.13	8.30

As shown in Table 7, the confidence interval (CI) scores calculated for different evaluation criteria vary depending on the selected non-OP set. Notably, the model demonstrates consistently lower CI values when trained and evaluated using the biologically curated non-OP set ${\mathbb{N}}^{h}$, compared to the randomly non-OP set ${\mathbb{N}}^{r}$. The SimPep framework for OPS prediction, using ${\mathbb{N}}^{h}$ results in approximately 2% and 5% lower CIscores for specificity and sensitivity, respectively, than when using ${\mathbb{N}}^{r}$. Similarly, the SimPep framework for OPD prediction, ${\mathbb{N}}^{h}$ achieves a 1.5% lower CI for accuracy and specificity.

This observation suggests that the quality and relevance of the non-OP set have a direct impact on the model’s stability and confidence. The non-OP set ${\mathbb{N}}^{h}$, composed of peptides with biological relevance to the problem space, supports more consistent and reliable performance. In contrast, the ${\mathbb{N}}^{r}$ as the non-OP set, derived from random proteins with no known osteogenic involvement, introduces greater variability and uncertainty. The higher CI scores associated with ${\mathbb{N}}^{r}$ indicate that the model’s predictions are less stable on such unrelated sequences, likely due to their divergence from the feature space learned during training.

### 3.7. Comparison of SimPep framework and baseline machine learning models

To assess the importance of SimPep’s architectural complexity for solving the OPD problem that detects osteogenic peptides, we compare its performance against three commonly used baseline machine learning models: RF, SVM, and XGBoost. These models are frequently applied in bioinformatics tasks and serve as relevant benchmarks. For a fair comparison, we use the same five-fold cross-validation strategy (see section 3.2.Five-fold cross-validation approach to make train and test sets) and identical data partitioning across all models.

**Fig 6:**
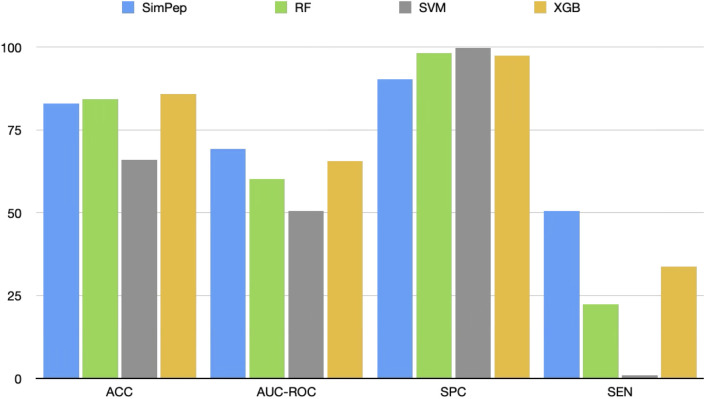
Benchmarking SimPep against RF, SVM, and XGBoost (XGB).

Each model is trained using the ProtBERT-derived representations of the peptides. Peptides labeled as osteogenic are assigned a label of 1, while non-osteogenic peptides are labeled as 0. The average performance across the five folds is reported in Fig 6.

Given the limited number of positive (osteogenic) samples, achieving high sensitivity is particularly important. SimPep demonstrates a significant advantage over the baseline models. Specifically, it achieves approximately 9%, 19%, and 4% higher AUC-ROC scores compared to RF, SVM, and XGBoost, respectively. More notably, SimPep outperforms all three models in terms of sensitivity, with improvements of approximately 23%, 50%, and 17% over RF, SVM, and XGBoost, respectively.

In addition to the baseline models, it is important to consider comparisons with other state-of-the-art models in the field. However, to the best of our knowledge, SimPep is the first computational framework specifically designed to predict the osteogenic potential of peptides. Existing models such as CollaPPI [35], which have demonstrated strong performance in protein-related prediction tasks, are typically based on graph neural networks and rely on 3D structural information. These approaches share certain architectural principles with SimPep, such as multi-branch representations.

Despite the advantages of CollaPPI, the application of its structure-based model to peptides is limited by the availability and reliability of 3D structural data. Unlike full-length proteins, short peptides often lack stable and well-defined tertiary structures in isolation, making the generation of consistent 3D input data challenging [36–38]. As a result, while models like CollaPPI are highly effective for larger protein systems, they are not directly applicable to short peptide sequences.

### 3.8. Evaluating SimPep framework on the external test sets

To further evaluate the reliability of our model, we apply two separate external peptide sets. The first, the external set, consists of OPs identified between 2022 and 2024. The second, the external set, includes peptides that were previously identified experimentally as non-OPs in our earlier research [22].

#### 3.8.1. External test positive peptide set.

We select the known OPs sourced from peer-reviewed articles published between 2022 and 2024 as the OP external test set comprising 26 peptides, defined as γ⊆𝕆. As mentioned earlier, PeptideRanker [39] is a powerful tool for predicting the bioactive peptides. To do a preprocess on the γ, we utilize this tool to determine the bioactivity of the peptides. According to the conducted virtual screening, the PeptideRanker [39] analysis shows that 14 out of 26 peptides are known as non-bioactive and 12 as bioactive. We define a positive training dataset O, with OPs identified prior to 2022 (O⊆𝕆). The set ${\mathbb{N}}^{h}$ including 488 non-OPs as the negative training set. The SimPep framework is performed with three inputs O, ${\mathbb{N}}^{h}$ and γ. Our framework accurately identified 10 out of 12 bioactive peptides as osteogenic. Our analysis reveals that SimPep not only successfully predicts osteogenic peptides but also discerns bioactive candidates, effectively filtering out non-active suggestions. This underscores the robustness and efficacy of the proposed model. The more details are available in Table 8.

**Table 8:** The OPD prediction score ($\varrho_{P}=OP-Pre(\gamma ,O,{\mathbb{N}}^{h})$ in the seventh step of the SimPep framework) on external osteogenic peptides published between 2022 and 2024. Bioactivity score ($\beta_{P}$) is computed by PeptideRanker [39].

Peptide Sequence (P)	$\beta_{P}\left(\%\right)$	$\varrho_{P\;}\left(\%\right)$	Ref.	Peptide Sequence (P)	$\beta_{P}\left(\%\right)$	$\varrho_{P\;}\left(\%\right)$	Ref.
GPAGPHGPVG	**82.52**	**95.23**	[40]	FDNEGKGKLPEEY	14.39	11.33	[41]
APDPFRMY	**94.17**	45.47	[40]	FWDGRDGEVDGFK	45.46	**83.27**	[41]
TPERYY	19.91	13.82	[40]	VLQTDNDALGKAK	15.5	13.62	[41]
IERGDVVVQDSPSD	5.14	10.59	[42]	IVLDSGDGVTH	11.51	13.83	[41]
RGDLGIEIPTEK	13.49	14.97	[42]	MVAPEEHP	12.53	12.66	[41]
YLLF	90.37	12.27	[43]	TWWNPRLVYFDY	**52.07**	**59.61**	[44]
YVEEL	6.28	11.11	[43]	QHREDGS	8.6	12.04	[45]
WWHS	**91.34**	**90.24**	[46]	MNKKREAEFQ	9.2	18.94	[47]
WWHJ	**97.51**	**94.65**	[46]	DEDEQIPSHPPR	38.69	13.91	[48]
WWHP	**98.82**	**92.63**	[46]	RVYFFKGKQYWE	35.11	13.28	[49]
WWHO	**97.51**	**94.51**	[46]	FGL	**97.2**	**77.22**	[50]
WWHD	**94.9**	**96.87**	[46]	GPO	**83.55**	**52.14**	[51]
WWHE	**84.87**	**96.15**	[46]	MGTSSTDSQQAQHRRCSTSN	8.56	12.63	[51]

#### 3.8.2. External test negative peptide set.

In the previous study [22], three peptides ‘VQSRYPSY’, ‘YPPQVMQY’, and ‘KIEEQQQTEDEQQDKIY’ were experimentally found out as non-OPs. We define γ includes these peptides. The molecular weight, net charge, and solvent of these peptides are available in columns number 2–4 in Table 9.

**Table 9:** The OPD prediction ($\varrho_{P}=OP-Pre(\gamma ,𝕆,{\mathbb{N}}^{h})$ in the seventh step of the SimPep framework) on external non-osteogenic peptides published in [22]. Bioactivity score ($\beta_{P}$) is computed by PeptideRanker [39].

Peptide Sequence (P)	Molecular weight	Net charge at pH 7.0	Solvent	$\beta_{P}\left(\%\right)$	$\varrho_{P\;}\left(\%\right)$
VQSRYPSY	999.0930	1	Distilled Water	22.04	19.57
YPPQVMQY	1025.189	0	Distilled Water	47.40	17.55
KIEEQQQTEDEQQDKIY	2152.257	-4	Distilled Water + Ammonia solution 25%	4.21	17.10

We perform the SimPep framework on the OP set, 𝕆, and non-OP set, ${\mathbb{N}}^{h}$, as the positive and negative training datasets and the test peptide set, γ. Notably, SimPep accurately predicts them as non-OPs. The predicted osteogenic likelihood for these peptides is available in the sixth column of Table 9. The comparison between these predictions and experimental results further underscores the efficacy of our model in discerning non-OPs. Consequently, our model exhibits a limited false positive rate, highlighting its reliability and potential utility in biomedical research and drug discovery endeavors.

### 3.9. Case study

The results obtained in the preceding sections highlight the efficacy of our proposed framework, SimPep, in accurately predicting OPs. This model holds promise for aiding in the discovery of novel peptides with osteogenic properties.

The previous research [20] has demonstrated the potential osteogenic activity of casein hydrolysates under 10 kDa, by lysing camel milk proteins using chymotrypsin [20]. According to the findings in this study, all four types of casein (alpha s1, alpha s2, beta, and kappa) found in camel, bovine, and human milk are selected from UniProt [24] to prepare for making the case study set. Then, PeptideCutter [25] is employed to predict cleavage sites in caseins using the high-specificity enzyme chymotrypsin. Utilizing this approach, 132 peptides are selected as the case study set shown by $\gamma =\{P_{1}^{\gamma },...,P_{132}^{\gamma }\}$.

In this section, we aim to leverage the predictive capabilities of the SimPep framework to identify potential osteogenic peptides obtained from caseins listed in γ. To accomplish this, we perform the SimPep framework on the three parameters: the positive training set 𝕆, the negative training set ${\mathbb{N}}^{h}$, and the test peptide set γ.

As the framework may have variations in training across different runs, we repeat the prediction process 10 times to enhance detection accuracy and extract the peptides deemed to have potential osteogenicity. Table 10 presents 16 selected peptides by our framework where $\varrho_{P\text{\textbackslash{} }}\geq \text{08}$. This threshold was chosen to ensure that the subsequent steps focus on peptides with a higher potential of being OPs.

**Table 10:** The list of potential osteogenic peptides derived from casein types using SimPep framework where $\varrho_{P\;}\geq 0.8$ ($\varrho_{P}=OP-Pre(\gamma ,𝕆,{\mathbb{N}}^{h})$ in the seventh step of the framework). N shows the number of preformation of the framework out of 10 where $\varrho_{P\;}\geq 0.8$.

Abb.	Peptide name	Peptide sequence (P)	N	Average $\varrho_{P\;}\left(\%\right)$
P1	camel-alpha s1-peptide 1	MKLLILTCLVAVALARPKYPLRYPEVF	5	88.23
P2	camel-alpha s1-peptide 8	HLEPFPQF	8	84.11
P3	camel-alpha s2-peptide 8	DQGKTRAYPF	2	82.39
P4	camel-beta-peptide 5	SHTEPIPYPILPQNF	10	86.36
P5	camel-beta-peptide 9	QIPQPVPQTPMIPPQSLLSLSQF	2	83.12
P6	camel-beta-peptide 4	TFPQPQSLVY	1	85.06
P7	camel-kappa-peptide 2	LVVTILALTLPF	2	88.19
P8	bovin-alpha s1-peptide 1	MKLLILTCLVAVALARPKHPIKHQGLPQEVLNENLLRF	3	88.76
P9	bovin-beta-peptide 7	QEPVLGPVRGPFPIIV	4	81.80
P10	bovin-kappa-peptide 2	LVVTILALTLPF	2	88.19
P11	bovin-beta-peptide 4	LQPEVMGVSKVKEAMAPKHKEMPFPKYPVEPF	2	86.89
P12	human-alpha s1-peptide 8	VPFPPF	9	85.16
P13	human-beta-peptide 3	QPQPLIYPF	10	87.41
P14	human-beta-peptide 7	PQIPKLTDLENLHLPLPLLQPLMQQVPQPIPQTLALPPQPLW	9	84.42
P15	human-kappa-peptide 11	LPNSHPPTVVRRPNLHPSF	6	85.63
P16	human-alpha s1-peptide 1	MRLLILTCLVAVALARPKLPLRYPERLQNPSESSEPIPLESREEY	2	86.85

While the peptide P6 is reported in one experiment, it is excluded before advancing further. Moreover, it is imperative to validate these peptides through virtual screening. For a comprehensive investigation, we employ a five-step pipeline to validate and recommend candidate OPs as follows:

1. Selecting key receptors that are relevant to osteogenesis.
2. Docking the predicted peptides identified by SimPep as potential OPs to these receptors to assess their binding affinities.
3. Selecting the high bioactive peptides that exhibit strong binding to the receptors.
4. Selecting the non-toxic peptides.
5. Recommending a peptide for experimental test.

The details of each step are available in the following sub-sections.

#### 3.9.1. Selecting key receptors.

Signaling pathways such as Wnt, BMP, TGF-β, Hedgehog, PTH, FGF, Notch, and Hippo are essential for the differentiation of osteoblasts and the process of bone formation [52]. Based on [53], Wnt and BMP have been identified as two key signaling pathways in regulating osteogenic properties, as they play crucial roles in bone formation and the differentiation of osteoblasts. The receptors of these pathways (see Table 11) are known as Frizzled for Wnt signaling and bone morphogenetic protein receptors (BMPRs) for BMP signaling.

**Table 11:** The list of key receptors that are relevant to osteogenesis.

Receptor name	UniProt ID	Receptor name	UniProt ID
BMPR type-1A	P36894	Frizzled-4	Q9ULV1
BMPR type-1B	O00238	Frizzled-5	Q13467
BMPR type-2	Q13873	Frizzled-6	O60353
Frizzled-1	Q9UP38	Frizzled-7	O75084
Frizzled-2	Q14332	Frizzled-8	Q9H461
Frizzled-3	Q9NPG1	Frizzled-9	O00144
		Frizzled-10	Q9ULW2

#### 3.9.2. Molecular docking.

Here, we initiate molecular docking studies between osteogenic-related receptors and the identified peptides. To facilitate this, we employ HPEPDOCK [54], a specialized tool designed for protein-peptide docking simulations.

For docking analysis, we use the HPEPDOCK server to evaluate the potential interactions between selected peptides and osteogenic receptor proteins. We provide the server with FASTA sequences of the peptides and target proteins. When experimental structures are unavailable in the PDB file, HPEPDOCK internally generates 3D models using its integrated homology modeling tools [54]. The docking score output by HPEPDOCK estimates binding affinity based on a combined energy function incorporating van der Waals interactions, electrostatics, and desolvation energy. Since HPEPDOCK [54] is available for peptide sequences with a length of less than 30 amino acids, the peptides P8, P11, P14, and P16 are extracted from the suggestions. The rest of eleven peptides are docked to each receptor (see Table 11) solely.

**Fig 7:**
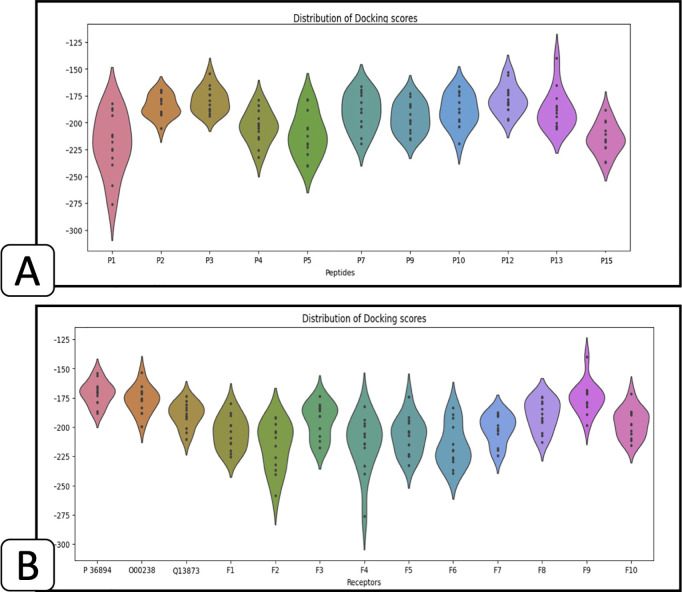
The distribution of docking scores. **(A)** Based on each peptide within 13 receptors, **(B)** Based on 11 peptides using 13 receptors.

Fig 7 depicts the distribution of docking scores for each peptide within 13 receptors. A more negative score correlates with a stronger binding affinity. Notably, peptides labeled as P1, P4, P5, and P15 demonstrate notably superior scores, as illustrated in Fig 7A.

Furthermore, we utilize violin charts to visually represent the prevalence of receptors within the receptor set that demonstrate effective docking with multiple peptides. As depicted in Fig 7B, Frizzled-2 (F2) and Frizzled-4 (F4) receptors emerge as particularly promising candidates for docking with osteogenic-like peptides, showcasing superior potential compared to other receptors. Frizzled-2 is participating in Wnt signaling pathway [55], which is crucial for bone formation and repair [56]. Similarly, Frizzled-4 is involved in canonical and non-canonical Wnt signaling [55]. Frizzled-4’s activation promotes osteogenic differentiation by regulating the expression of genes involved in bone formation and mineralization.

#### 3.9.3. Selecting bioactive peptides.

Subsequently, leveraging PeptideRanker [39], we predict the bioactivity of these peptides. This analysis discerns which peptides exhibit not only osteogenic properties but also broader bioactivity, thereby enriching our understanding of their therapeutic potential. According to PeptideRanker, peptide P1 with bioactivity 98.93%, exhibits notably higher levels of activation compared to P15 with 53.29% and P4 with 51.44%. However, P5 with a bioactivity of 12.69% is not bioactive.

#### 3.9.4. Toxicity analysis.

Lastly, we assess the toxicity properties of the peptides using ToxinPred2.0 [57], a tool proficient in predicting the toxicity of small proteins and peptides. This comprehensive approach ensures a thorough characterization of the identified peptides, facilitating informed decisions regarding their suitability for further experimental validation and potential therapeutic applications. According to the results, P15 is a toxin, P1 and P4 are non-toxins.

#### 3.9.5. Recommending a peptide for experimental test.

**Fig 8:**
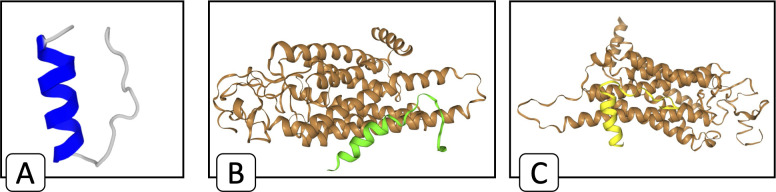
The docking of peptide P1 and the intended receptors. **(A)** The predicted structure of peptide P1using AAT Bioquest, **(B)** The docking of P1 peptide and Frizzled-2 receptor, **(C)** The docking of P1 peptide and Frizzled-4 receptor.

According to the corresponding results, peptides P1 and P4 are recommended for further investigation due to their osteogenic potential. However, since P1 exhibits better bioactivity compared to P4, we suggest prioritizing P1 = ‘MKLLILTCLVAVALARPKYPLRYPEVF’, as a potential osteogenic peptide that is both bioactive and non-toxic. This peptide is derived from camel milk alpha s1-casein, with a molecular weight of 3.12 kDa, calculated by AAT Bioquest [58]. Our previous research [20] has demonstrated that camel milk caseins, when lysed with chymotrypsin and reduced to a molecular weight of under 10 kDa, exhibit significant osteogenic properties. Numerous studies highlight the role of camel milk in promoting health and its potential to become a superfood due to its rich nutritional profile and health benefits, including its positive impact on bone health [59–61]. Moreover, the camel milk contains 4.9–5.7Ω alpha s1-caseins [62], which are involved in calcium-binding [63]. The predicted structure for P1 is illustrated in Fig 8A, predicted by PEP-FOLD 3 [64].

Moreover Fig 8B and 8C illustrate the docking conformations between the P1 peptide and the Frizzled-2 and Frizzled-4 receptors, respectively, as modeled by HPEPDOCK [54]. The docking scores reported by HPEPDOCK are -285.864 for the Frizzled-2 receptor and -300.363 for the Frizzled-4 receptor, indicating strong and favorable interactions in both cases.

## 4. Conclusion

This paper addressed the critical need for advancing computational approaches in the identification of OPs, which are essential for bone health, especially in the context of an aging population and the rising prevalence of osteoporosis. By creating the first publicly available database dedicated to experimentally validated OPs, named OP-AND, and proposing the novel hypothesis that peptides derived from proteins involved in osteoclast genesis are non-OP, this study fills a major gap in the current research landscape.

The development of the SimPep framework, utilizing deep learning models to predict OPs, represented a significant step forward in overcoming challenges posed by limited data availability and the absence of reliable negative peptide pools. The SimPep framework demonstrated strong performance, achieving an accuracy of 86.87% and an AUC-ROC of 76.88%, highlighting its effectiveness in detecting OPs from highly imbalanced datasets.

Through various experiments, including the validation of our non-OP hypothesis, cross-validation of OPD predictions, and a real-world case study involving casein types-derived peptides, this paper demonstrated applicability. In particular, the identification of a potential osteogenic peptide from alpha s1-casein in camel milk underscores the practical utility of the SimPep framework in discovering new bioactive peptides for experimental validation.

While the peptides included in the OP-AND database were curated based on literature reports claiming osteogenic activity, we acknowledge that future versions of this resource would benefit from more standardized inclusion criteria, such as quantitative ALP activity, mineralization levels, and consistent gene expression assays, to ensure uniform and rigorous biological validation across entries.

Furthermore, our results showed that the OPS task learned by SimPep-Net can meaningfully capture similarity between peptides with shared osteogenic potential. However, directly transferring OPS-derived outputs into OPD tasks may require further refinement. Future work may explore alternative architectures or hybrid training objectives to better leverage peptide similarity for robust OPD performance.

These findings collectively advance the field of computational osteogenic peptide discovery and provide a foundation for the development of more accurate, explainable, and scalable screening frameworks for bone-regenerative therapeutics.
